# Drug problems among homeless individuals in Toronto, Canada: prevalence, drugs of choice, and relation to health status

**DOI:** 10.1186/1471-2458-10-94

**Published:** 2010-02-24

**Authors:** Michelle N Grinman, Shirley Chiu, Donald A Redelmeier, Wendy Levinson, Alex Kiss, George Tolomiczenko, Laura Cowan, Stephen W Hwang

**Affiliations:** 1 Centre for Research on Inner City Health, The Keenan Research Centre in the Li Ka Shing Knowledge Institute of St. Michael's Hospital, Toronto, Canada; 2 Department of Medicine, University of Toronto, Toronto, Canada; 3 Sunnybrook Health Sciences Centre, Institute for Clinical Evaluative Sciences, Toronto, Canada; 4 Department of Research Design and Biostatistics, Institute for Clinical Evaluative Sciences, Sunnybrook Health Sciences Centre, Toronto, Canada; 5 Crohn's and Colitis Foundation of Canada, Toronto, Canada; 6 Department of Psychiatry, University of Toronto, Toronto, Canada; 7 Street Health Community Nursing Foundation, Toronto, Canada

## Abstract

**Background:**

Drug use is believed to be an important factor contributing to the poor health and increased mortality risk that has been widely observed among homeless individuals. The objective of this study was to determine the prevalence and characteristics of drug use among a representative sample of homeless individuals and to examine the association between drug problems and physical and mental health status.

**Methods:**

Recruitment of 603 single men, 304 single women, and 284 adults with dependent children occurred at homeless shelters and meal programs in Toronto, Canada. Information was collected on demographic characteristics and patterns of drug use. The Addiction Severity Index was used to assess whether participants suffered from drug problems. Associations of drug problems with physical and mental health status (measured by the SF-12 scale) were examined using regression analyses.

**Results:**

Forty percent of the study sample had drug problems in the last 30 days. These individuals were more likely to be single men and less educated than those without drug problems. They were also more likely to have become homeless at a younger age (mean 24.8 vs. 30.9 years) and for a longer duration (mean 4.8 vs. 2.9 years). Marijuana and cocaine were the most frequently used drugs in the past two years (40% and 27%, respectively). Drug problems within the last 30 days were associated with significantly poorer mental health status (-4.9 points, 95% CI -6.5 to -3.2) but not with poorer physical health status (-0.03 points, 95% CI -1.3 to 1.3)).

**Conclusions:**

Drug use is common among homeless individuals in Toronto. Current drug problems are associated with poorer mental health status but not with poorer physical health status.

## Background

Drug use is believed to be an important factor contributing to the poor health and increased mortality risk that has been widely observed among homeless individuals [1,2]. Substance use may increase the risk of homelessness by undermining their social ties and economic stability [3]. Drug users also suffer from numerous adverse health effects, including overdoses, psychiatric conditions, and infectious diseases [4,5].

Drug use patterns vary regionally and often change over time [6]. However, few recent studies in the peer-reviewed literature have examined patterns of drug use among homeless individuals in a major Canadian city. This study's goal was to determine the prevalence and characteristics of drug use among a stratified random sample of homeless individuals in Toronto. The prevalence of drug problems, as determined by the Addiction Severity Index, was also ascertained, and the association between drug problems and physical and mental health status was examined.

## Methods

### Sampling design

This study was part of a larger ongoing study of health care utilization among homeless individuals. For this reason, the study recruited a stratified random sample of homeless persons in Toronto in 2004-2005 who were registered with the province of Ontario's universal health insurance program [7]. Homelessness was defined as living within the last 7 days at a shelter, public place, vehicle, abandoned building, or someone else's residence, and not having a place of one's own. Approximately 90% of study participants were recruited at shelters and 10% at meal programs. These proportions were based on pilot data showing that 90% of homeless individuals in Toronto slept at shelters and 10% used meal programs but not shelters [8]. Recruitment was stratified to obtain a 2:1:1 ratio of males without dependent children, females without dependent children, and adults accompanied by dependent children to ensure adequate power for comparisons among these three groups. When a family with dependent children included two or more adults, one adult was randomly selected for inclusion in the analysis.

Permission to recruit participants was granted by 58 (91%) of 64 shelters in Toronto for men, women, youth, and families. Recruitment also took place at 18 meal programs randomly selected from 62 sites serving homeless people. The number of participants recruited at each site was proportionate to the number of homeless individuals served monthly at that site. Participants were selected from bed lists or meal lines using a random number generator and then screened for eligibility. Because the goal of recruiting at meal programs was to enroll homeless people who did not use shelters, we excluded individuals at meal programs who had used a shelter within the last 7 days.

Of 2,516 individuals screened at homeless shelters and meal programs, 882 individuals (35%) were ineligible for the following reasons: 229 (9%) did not meet our definition of homelessness, 104 (4%) were unable to communicate in English, 54 (2%) were homeless shelter users encountered at meal programs, 53 (2%) were unable to give informed consent, and 442 (18%) were excluded because they did not have an Ontario health insurance number. A health insurance number was required to allow tracking of participants' use of the health care system, since this was the main goal of the primary study of which this was a part. Those excluded due to lack of a health insurance number consisted mainly of refugees, refugee claimants, or recent migrants to Ontario. Of 1,634 eligible individuals, 443 declined to participate and 1,191 individuals (73% of those eligible) were enrolled in the study. Each participant provided written informed consent and received $15 for completing the survey. This study was approved by the research ethics board at St. Michael's Hospital in Toronto, Canada.

### Survey instrument

Demographic characteristics were obtained by self-report. Race/ethnicity was self-identified, with participants selecting from categories from the Statistics Canada Ethnic Diversity Survey [9]. Participants identified the first and second most important factors that they felt were keeping them from getting out of homelessness. Their free responses were coded into mutually exclusive categories, including lack of employment, lack of suitable housing, and addiction to drugs and/or alcohol.

Details were obtained about drug use, including the types of substances ever used, how recently each drug had been used, and frequency of use in the last 30 days. We differentiated between drug use and drug problems. The Addiction Severity Index (ASI), which identifies individuals whose daily functioning is affected by their drug use, was used to determine whether participants had current drug problems (ie. within the last 30 days) [10,11]. For example, the ASI includes questions regarding the effects of drug use on physical health (such as adverse drug reactions or black outs), social interactions (such as loss of friends or neglect of family), and integration into society (such as missed work or illegal activities) [10]. This instrument has been validated in studies of homeless individuals [12-14]. Drug problems were dichotomized as present or absent based on criteria previously used with homeless populations [15]. The ASI was also used to determine whether participants had mental health problems or alcohol problems in the last 30 days [10,11].

The SF-12, a widely used general health status instrument that has been validated in homeless populations, [16,17] was used to generate measures of physical health status (physical component subscale (PCS)) and mental health status (mental component subscale (MCS)). These scores range from 13 to 69 for physical health and 10 to 70 for mental health, standardized to a mean of 50 and standard deviation of 10 in the general population in the United States [17].

### Statistical analyses

Data analyses were performed using SPSS 16.0. Prevalence of drug use was classified as recent (within the past 2 years) or remote (>2 years ago). For each drug type, the median number of episodes of use in the past 30 days was calculated. Characteristics of participants with and without drug problems were compared using chi-square and t-tests. Linear regression models were constructed to determine if current drug problems were associated with physical health status or mental health status, after adjustment for age, sex, race/ethnicity, and level of education.

## Results

Lifetime prevalence of regular use of at least one drug was reported by 712 individuals (60%). In contrast, current drug problems (i.e., within the last 30 days) were present in 475 individuals (40%). The prevalence of current drug problems varied widely by sex and family status: 53% among single men, 41% among single women, and 12% among adults accompanied by dependent children. A comparison of characteristics of individuals with and without a current drug problem revealed numerous differences (Table 1). Individuals with current drug problems were significantly more likely to be single men, white, Canadian-born, and lacking a high school degree. They were also more likely to be younger and to have become homeless at a younger age. Participants with current drug problems had a longer lifetime duration of homelessness (mean 4.8 vs. 2.9 years, p < 0.001). Alcohol problems within the last 30 days were present among 45% of individuals with current drug problems, compared to 19% among those without current drug problems (p < 0.001). Among individuals with drug problems in the last 30 days, only 27% identified drug and/or alcohol use as a factor keeping them from getting out of homelessness.

**Table 1:** Characteristics of study participants. Data are given as numbers (%), unless otherwise specified.

	All participants (N = 1191)	With current drug problem (n = 475)	Without current drug problem (n = 716)	p-value
Age, mean (SD)	36.2 (12.3)	34.2 (11.1)	37.5 (12.9)	< 0.001

Sex/family status				< 0.001
Single male	603 (50.6)	317 (66.7)	286 (39.9)	
Single female	304 (25.5)	125 (26.3)	179 (25.0)	
Adult accompanied by children	283 (23.7)	33 (6.9)	250 (34.9)	

Race/ethnicity				< 0.001
White	662 (55.6)	300 (63.2)	362 (50.6)	
Black	266 (22.3)	72 (15.2)	194 (27.1)	
First Nations	100 (8.4)	60 (12.6)	40 (5.6)	
Other race/ethnicity	163 (13.7)	43 (9.1)	120 (16.8)	

Region of birth				< 0.001
Canada	812 (68.2)	392 (82.5)	420 (58.7)	
Outside Canada	379 (31.8)	83 (17.5)	296 (41.3)	

Education				< 0.001
Some high school or less	597 (50.3)	290 (61.3)	307 (42.9)	
High school or equivalent	255 (21.5)	92 (19.5)	163 (22.8)	
Vocational training/college or above	336 (28.3)	91 (19.2)	245 (34.3)	

Age at first episode of homelessness, mean (SD)	28.4 (13.0)	24.8 (11.9)	30.9 (13.2)	< 0.001

Duration of current episode of homelessness in months, mean (SD)	15.7 (35.2)	18.7 (37.2)	13.8 (33.7)	0.02

Lifetime years of homelessness in years, mean (SD)	3.7 (5.5)	4.8 (5.6)	2.9 (5.2)	< 0.001

Drugs and alcohol reported as an impediment from getting out of homelessness	162 (13.6)	128 (26.9)	34 (4.7)	< 0.001

Simultaneous use of >1 drug in past month*	501 (42.1)	363 (76.4)	138 (19.3)	< 0.001

Mean pack-years smoked (SD)	17.8 (29.6)	21.5 (24.6)	15.4 (32.3)	< 0.001

Mental health problems (present within the last 30 days)	444 (37.3)	212 (44.6)	232 (32.4)	< 0.001

* This question was only asked of participants who had ever used drugs in their lifetime.

**Figure 1:**
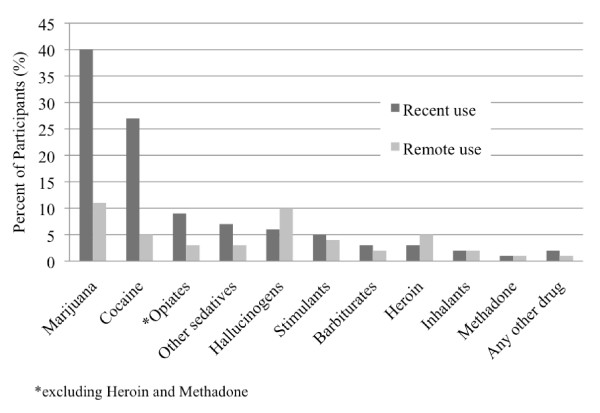
**Prevalence of drug use according to type of substance and recent use (within the past 2 years) versus remote use (more than 2 years ago) (n = 1191)**.

Prevalence of drug use is shown in Figure 1, by specific substance. The substances most commonly used within the past 2 years were marijuana (40%), cocaine (27%), and opiate analgesics other than heroin or methadone (i.e., oxycodone, hydromorphone, meperidine, codeine, and pentazocine) (8%). Drug use within the past 2 years varied by demographic group, with marijuana use reported by 51% of single men, 35% of single women, and 21% of adults with dependent children (p < 0.001). Cocaine use was 32%, 32%, and 9%, respectively (p < 0.001). Opiate use was 11%, 8%, and 4%, respectively (p = 0.016).

**Figure 2:**
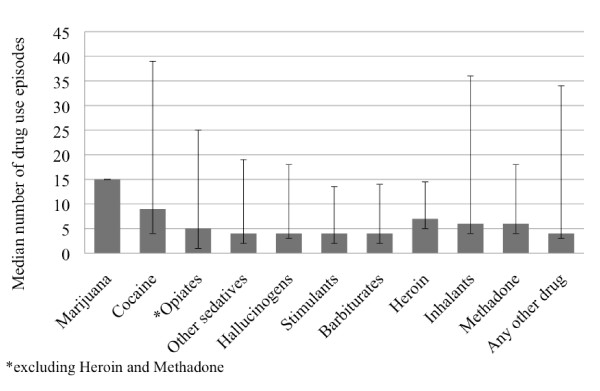
**Intensity of drug use within the past 30 days represented as mean number of episodes of drug use with 25^th ^and 75^th ^quartiles for each drug category**.

Figure 2 shows the median number of days of drug use within the past 30 days, among individuals reporting any use during that time frame. Marijuana and cocaine had the highest reported use in the last 30 days (median of 15 and 9 episodes, respectively). In contrast, heroin, methadone and other opiates were used a median of 7, 6, and 5 episodes in the past 30 days, respectively.

Physical health status and mental health status, as measured by the SF-12, are shown in Table 2. In univariate analysis, drug problems were associated with significantly lower mental health status. In linear regression models adjusted for age, sex, level of education, and race/ethnicity, having current drug problems was associated with significantly poorer mental health (MCS -4.9 points, 95% CI -6.5 to -3.2) but not with poorer physical health (PCS -0.03, 95% CI -1.3 to +1.3). A similar pattern was observed in models examining the association between cocaine use and mental and physical health scores (MCS -4.7 points, 95% CI -6.4 to -3.0; PCS -1.3 points, 95% CI -2.7 to +0.06).

**Table 2:** Associations between current drug problem and health status, as measured by SF-12 scores.

	Physical Component Subscale	Mental Component Subscale
	**Mean (SD)**	**Mean (SD)**

Current drug problem	46.6 (10.6)	38.8 (12.9)
Without current drug problem	45.7 (11.4)	42.0 (13.2)
*p-value*	0.17	< 0.001

Recent cocaine use	44.8 (11.4)	38.1 (12.7)
No recent cocaine use	46.5 (11.0)	41.7 (13.2)
*p-value*	0.02	< 0.001

	Beta (95% CI)	Beta (95% CI)

Change in SF-12 score associated with		
Current drug problem*	-0.03 (-1.3 to +1.3)	-4.9 (-6.5 to -3.2)
Recent cocaine use*	-1.3 (-2.7 to +0.06)	-4.7 (-6.4 to -3.0)

* In linear regression models adjusted for sex, age, level of education, and race/ethnicity.

## Discussion

The prevalence of drug use was found to be very high among homeless individuals in Toronto compared to rates previously reported for the general population. Lifetime drug use among participants was 60%; in contrast, a study by Vega and colleagues (2002) on the prevalence of drug use at 7 international sites found rates of 32% for men and 20% for women in the province of Ontario [18]. A similar pattern was observed for the use of specific drugs. The prevalence of cocaine use within the past 2 years among study participants (27%) was 27-fold higher than the corresponding figure of 1% in the general Canadian population, as reported by the Toronto Drug Strategy Advisory Committee (2005) [19]. Regular marijuana use (40%) was almost three times higher than that found in the general population (14%) in the 2006 Canadian Addiction Survey [20].

High rates of drug use among homeless people result from a number of processes. Drug use is a risk factor for becoming homeless [21]. Drug use is also a risk factor for prolonged homelessness [22], which is confirmed by our data. As a result of this association, drug users are over-represented in cross-sectional surveys of homeless populations [23]. Homelessness may increase the likelihood that an individual will use drugs.

However, only about one-quarter of participants with current drug problems identified drug and/or alcohol use as an impediment to acquiring stable housing, which is consistent with a recent survey of 368 homeless individuals in Toronto in which only 23% of participants identified their drug or alcohol use as the main reason for becoming homeless [24]. Some homeless individuals may be in a state of denial or lack insight regarding the impact of their substance use. Alternatively, this finding may reflect the multifactorial causes of homelessness. Either of these possibilities has practical implications for assisting homeless individuals with drug problems. There is controversy regarding the effectiveness of adopting a "housing first" approach versus interventions that require substance abuse treatment and/or abstinence from drug use as a pathway to obtaining stable housing [25].

As expected, drug problems were associated with significantly poorer mental health status. The lack of association between drug problems and physical health status is surprising given the many physical health complications related to drug use [4,5]. This finding may be explained by a long latency period between the initiation of drug use and deterioration in physical health, survival bias due to the death of drug users with poor physical health, or selection bias due to the exclusion of hospitalized individuals. Nonetheless, our findings suggest that service providers should recognize that mental health, rather than physical health, may represent the greatest challenge for homeless individuals with drug problems.

This study has a number of strengths. A large stratified random sample of homeless men and women, including both shelter and non-shelter users, were recruited across numerous community sites in a major North American city. The recruitment rate of eligible individuals was very high at 73%. Also, the ASI instrument utilized in this study has been previously validated among homeless people [12-14].

This study has a few limitations. There was no control group of non-homeless individuals. The exclusion of individuals who were unable to give informed consent may have resulted in the elimination of people who were under the influence of drugs. The requirement that study participants have a health insurance number resulted in the exclusion of refugees and refugee claimants. Lastly, drug use was assessed on the basis of self-report. While this method is subject to recall errors and social desirability bias, it has been validated as a fairly accurate measure among homeless individuals [12].

## Conclusions

Our study demonstrates the high prevalence of drug use among the homeless population of Toronto. Drug use is associated with a substantial negative impact on mental health, as well as earlier onset and longer duration of homelessness. These findings suggest the need for early interventions aimed at preventing initiation of street drug use. Improved access to drug treatment programs for homeless individuals is also needed.

## Competing interests

The authors declare that they have no competing interests.

The funders for this study did not play any role in the study design, in the collection, analysis, and interpretation of data; in the writing of the report; and in the decision to submit the article for publication. The views expressed in this publication are the views of the authors and do not necessarily reflect the views of the Ontario Ministry of Health and Long-Term Care or any of the other organizations that have been acknowledged.

## Authors' contributions

SC, DAR, WL, AK, GT, LC, and SWH contributed to the study concept and design, and critically reviewed the manuscript for important intellectual content. MNG performed the statistical analysis, data interpretation, as well as the drafting and critical review of the manuscript for important intellectual content. SC oversaw the study data collection, and conducted the statistical analysis. SWH supervised the overall study and the statistical analysis, and interpreted the data. All authors reviewed the manuscript personally and agreed to its publication.

## Pre-publication history

The pre-publication history for this paper can be accessed here:

http://www.biomedcentral.com/1471-2458/10/94/prepub
